# Importance of Gene Duplication in the Evolution of Genomic Imprinting Revealed by Molecular Evolutionary Analysis of the Type I MADS-Box Gene Family in *Arabidopsis* Species

**DOI:** 10.1371/journal.pone.0073588

**Published:** 2013-09-05

**Authors:** Takanori Yoshida, Akira Kawabe

**Affiliations:** Faculty of Life Science, Kyoto Sangyo University, Kyoto, Kyoto, Japan; CNRS, France

## Abstract

The pattern of molecular evolution of imprinted genes is controversial and the entire picture is still to be unveiled. Recently, a relationship between the formation of imprinted genes and gene duplication was reported in genome-wide survey of imprinted genes in *Arabidopsis thaliana*. Because gene duplications influence the molecular evolution of the duplicated gene family, it is necessary to investigate both the pattern of molecular evolution and the possible relationship between gene duplication and genomic imprinting for a better understanding of evolutionary aspects of imprinted genes. In this study, we investigated the evolutionary changes of type I MADS-box genes that include imprinted genes by using relative species of *Arabidopsis thaliana* (two subspecies of *A* *. lyrata* and three subspecies of *A* *. halleri* ). A duplicated gene family enables us to compare DNA sequences between imprinted genes and its homologs. We found an increased number of gene duplications within species in clades containing the imprinted genes, further supporting the hypothesis that local gene duplication is one of the driving forces for the formation of imprinted genes. Moreover, data obtained by phylogenetic analysis suggested “rapid evolution” of not only imprinted genes but also its closely related orthologous genes, which implies the effect of gene duplication on molecular evolution of imprinted genes.

## Introduction

Genomic imprinting is a phenomenon that causes complete or partial uniparental gene expression of particular genes (called “imprinted genes”). In plants, genomic imprinting mainly occurs in the endosperm of developing seeds that nourish the embryo during and after seed development. Recent studies using genome-wide surveys have identified more than one hundred candidate imprinted genes in *Arabidopsis thaliana*, *Oryza sativa*, and *Zea mays* [1–6].

The basis of regulation of genomic imprinting in developing seeds has been intensively investigated, and two epigenetic mechanisms have been identified as factors that regulate genomic imprinting in plants: (1) DNA methylation and (2) histone methylation [7–12]. In plants, the methylation status of the differently methylated regions in the proximal region of the imprinted genes between maternal and paternal alleles affects the expression of some imprinted genes [7,8]. In addition to the methylation status of the DNA, trimethylation of H3K27 catalyzed by Polycomb repressive complex 2 (PRC2) also affects the expression of imprinted genes by silencing either the paternal or maternal allele [12]. It is reported that transposable elements (TEs) are methylated in the embryo but extensively demethylated in the endosperm, affecting the imprinting status of the nearby genes [1]. These results may support the defense theory that the status of imprinting arises as a byproduct of silencing of the invading DNA fragments such as TEs [13]. Recent studies carried out using genome-wide analysis of imprinted genes have focused on understanding the relationship between gene duplication and the gene imprinting status [4]. Together with TEs, gene duplication might also affect the imprinting status and evolution of imprinted genes.

Another question concerning genomic imprinting is the pattern of molecular evolution of the imprinted genes. The parental conflict theory predicts that conflict between maternal and paternal genomes causes evolutionary “arms races” and that the imprinted genes show accelerated molecular evolution [14]. For example, Spillane et al. suggested that an imprinted locus *MEDEA* in *Arabidopsis* is under the influence of positive Darwinian selection with neo-functionalization after its generation by whole-genome duplication [15]. However, in the analysis of molecular evolution of some imprinted genes of both mammals and plants, no evidence for antagonistic coevolution was detected [16,17]. Thus, a comprehensive understanding of the effect of genomic imprinting on the pattern of molecular evolution of imprinted genes remains elusive.

In this study, we investigated the evolutionary changes of type I MADS-box genes, which include imprinted genes, by using relative species of *A. thaliana*. MADS-box genes encode transcription factors that contain the conserved MADS-box domain. Members of type I MADS-box gene family in plants have been reported to be involved in reproductive development and expressed in developing seeds (Gene networks in seed development website; available from: http://seedgenenetwork.net/) [18,19]. Some genes in the family (*PHERES1*, *At1g59930*, *AGL36*, and *AGL92*) show imprinted gene expression in *A. thaliana* [3,20,21]. *PHERES1* and *AGL36* were identified as imprinted genes by the observation of expression patterns in the endosperms [20–22]. *At1g59930* and *AGL92* were identified by deep sequencing of the developing seeds [3]. *PHERES1* and *AGL92* are paternally expressed, whereas *AGL36* and *At1g59930* are maternally expressed. The available data suggest that in both *O. sativa* and *A. thaliana* type I MADS-box, gene duplications occurred independently [23]. One of the duplicated genes, *OsMADS87*, is also reported as a maternally expressed imprinted gene [24]. Imprinted genes of these two species emerged independently among duplicated gene clusters. Unlike single-copy imprinted genes, duplicated genes enable us to compare DNA sequences of imprinted genes and their non-imprinted homologs. Thus, type I MADS-box genes are a useful resource to study molecular evolution of imprinted genes and the relationship between gene duplication and genomic imprinting. First, we focused on identifying the relationship between gene duplication and the genomic imprinting status. The incidence of gene duplication in each clade of type I MADS-box genes was estimated to assess its relationship to genomic imprinting. Next, we investigated the effect of genomic imprinting on molecular evolution. Our results suggest a positive relationship between gene duplication and the incidence of imprinted genes and “rapid evolution” in clades containing imprinted genes. We discuss possible driving forces that trigger these correlations, thereby bringing about the evolution of imprinted genes.

## Materials and Methods

### Plant materials

**Figure 1:**
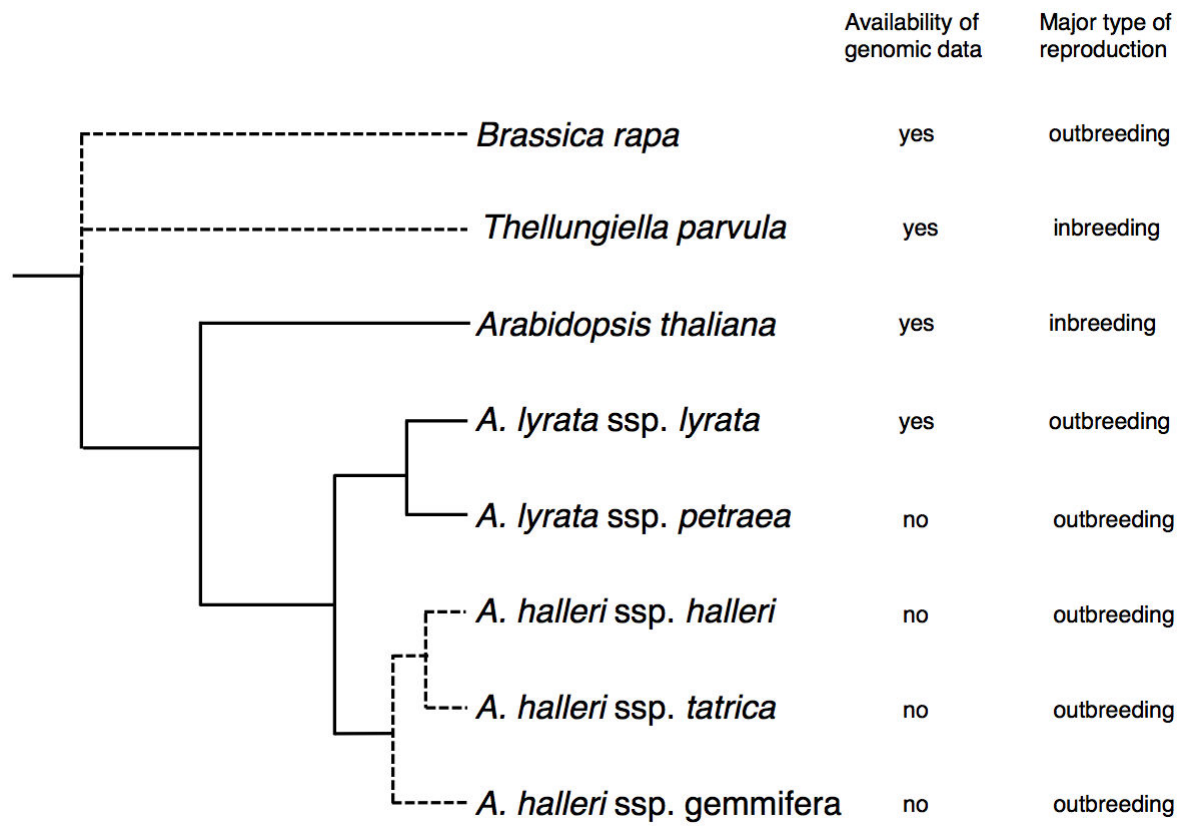
Schematic diagram showing the phylogenetic relationship among species used in this study. The reproduction type and availability of genomic data are also indicated. Dashed lines are used to indicate partly ambiguous phylogenetic relations.

Closely related species of *A. thaliana* were used in this study (Figure 1). Publicly available genomic data of *A. thaliana*, *A* *. lyrata* ssp. *lyrata* , *Thellungiella* *parvula* (salt cress), and *Brassica* *rapa* were used in this study for comparisons [25–28]. The origin of other plant materials was as follows: four *A* *. lyrata* ssp. *petraea* strains from Plech Germany (kindly provided by J. de Meaux); *A. halleri* ssp. *gemmifera* strain 144-1 isolated in Mino-shi, Osaka, Japan; *A* *. halleri* ssp. *tatrica* strain T-PLDH1 isolated in Vysoke Tatry, Poland; *A* *. halleri* ssp. *halleri* strain H–RB isolated in Bistrita, Romania; *Turritis* *glabra* strain OM isolated in Ohmi-Shirahama, Shiga, Japan; and *Crucihimalaya* *wallichii* strain SJS00500 obtained from RIKEN BioResource Center.

### Sequence analysis of orthologous type I *MADS*-box genes from a publicly available database

Sequences of type I MADS-box genes of *A. thaliana*, *A* *. lyrata* ssp. *lyrata* , *T* *. parvula* , and *B* *. rapa* were collected from a publicly available NCBI Genome database (http://www.ncbi.nlm.nih.gov/genome). For *A* *. lyrata* ssp. *lyrata* , *T* *. parvula* , and *B* *. rapa* , we performed a BLAST search optimized for somewhat similar sequences (blastn) to search database of each genome with sequences of *At5g26575* (*AGL34*), *At5g26630* (*AGL35*), *At5g26650* (*AGL36*), *At1g65330* (*PHE1*), *At1g65300* (*PHE2*), *At3g05860* (*AGL45*), *At2g28700* (*AGL46*), *At5g48670* (*AGL80*), *At1g31630* (*AGL86*), *At5g27960* (*AGL90*), and *At1g31640* (*AGL92*) as queries. We also used MADS-box family genes *At5g35120*, *At1g59920*, and *At1g59930* that lack the MADS-box motif.

### Cloning and sequencing of type I MADS-box genes

In this study, we analyzed type I MADS-box genes in *A. thaliana* because these genes contain several imprinted genes and are phylogenetically related to each other. To obtain orthologous genes of each type I MADS-box gene of *A. thaliana* from relative species, eight primer pairs were used for PCR (Table S1 in File S1). Genomic DNAs were isolated from fresh leaves by using DNeasy Plant Mini Kit (QIAGEN, USA). DNA fragments orthologous to type I MADS-box genes were amplified by PCR and cloned using T-Vector pMD20 (TaKaRa, Japan) and Ligation Mighty Mix (TaKaRa, Japan). Multiple DNA sequences from *A* *. lyrata* ssp. *petraea* and three subspecies of *A* *. halleri* (ssp. *halleri*, ssp. *tatrica*, and ssp. *gemmifera*) were cloned. For each primer pair, we sequenced up to 15 clones from each relative species. Only sequences that were verified at least twice from independent clones were used for the following analyses. When diverged sequence(s) was obtained from the initial 15 clones, additional clones were sequenced. The sequence data was aligned by manual inspection. Newly determined sequences were deposited in DDBJ under the accession numbers AB830633-AB830706.

### Computational analyses of molecular evolution

Phylogenetic relationship was estimated by the neighbor-joining method with Jukes and Cantor distance [29] by using the MEGA5 program [30]. Pairwise ω (*d*_N_/*d*_S_) ratios of type I MADS-box genes and other MADS transcription factor family genes between *A. thaliana* and *A* *. lyrata* ssp. *lyrata* were calculated using DnaSP ver.5 [31]. BLAST searches were performed to find pairwise genes between *A. thaliana* and *A* *. lyrata* ssp. *lyrata* . Annotated *A. thaliana*’s 46 type I MADS-box genes and 63 other MADS transcription factor family genes (TAIR *Arabidopsis* Gene Family Information; available from: http://www.arabidopsis.org) were used as queries. For each clade of type I MADS-box gene, we estimated the ratio of non-synonymous (*d*_N_) to synonymous (*d*_S_) substitution rate (*ω*) by the maximum likelihood method implemented in program codeml in program package PAML ver. 4.4b [32]. A sequence of *A* *. lyrata* that showed similarity to the *At1g59930* gene was excluded from the analysis because of the large deletion. Sequences from *T* *. glabra* were used as out-groups. We could not obtain the *T* *. glabra* orthologous sequence of *AGL46*; therefore, the sequence obtained from *C* *. wallichii* was used as an out-group. Tree topologies of each clades constructed by the neighbor-joining method were used in simulations of codeml. We applied a free-ratio model in which it is assumed that *n* parameters of the ω-ratio are equal to the total number of branches in the phylogeny.

Our data suggested that branches in the clade containing imprinted genes have evolved faster (higher ω-ratio) than those in the clade without imprinted genes. To verify this tendency, we estimated ω-ratio using the following models: Model 0, one-ratio model with single ω-ratio for all branches and Model 1, two-ratio model with two ω-ratios (*ω*_1_ and *ω*_2_). A likelihood ratio test between Model 0 and Model 1 was conducted.

## Results

### Phylogenetic analyses of orthologous type I *MADS*-box genes

**Figure 2:**
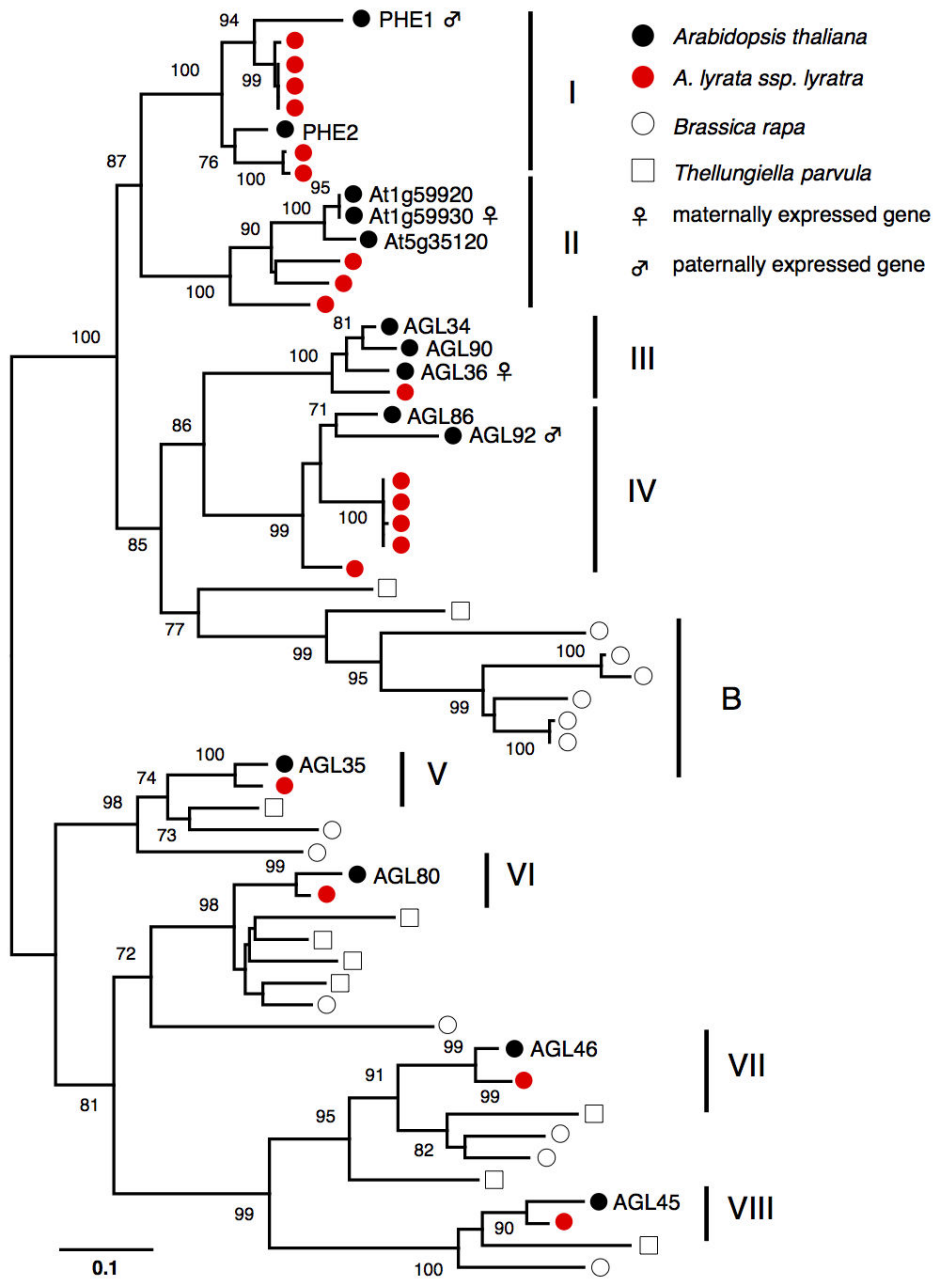
Neighbor-joining tree of type I MADS-box genes. Black circles; *A*. *thaliana*, red circles; *A*. *lyrata* ssp. *lyrata*, empty circles; *B*. *rapa*, and empty squares; *T*. *parvula*. Bootstrap values (%) were estimated by 500 replications for each clade and shown at corresponding nodes. Nine distinguishable clades shown as thick vertical lines are designated as clade I–VIII and clade B. The scale bar is shown below the tree.

The phylogenetic relationship of the relative species of *A. thaliana* used in this study is shown in Figure 1. We used gene sequences from the whole-genome sequences of *A. thaliana*, *A* *. lyrata* ssp. *lyrata* , *T* *. parvula* , and *B* *. rapa* to estimate phylogenetic clustering and the number of duplications within and among species. Phylogenetic relationships of type I MADS-box genes close to known imprinted genes were estimated by the neighbor-joining method (Figure 2). We identified eight distinguishable clades (designated as clades I–VIII in Figure 2) when *A. thaliana* and *A* *. lyrata* genes were considered. Each clade from I to IV contained one imprinted gene, whereas there were no imprinted genes in clades V–VIII. Thus, we compared clades I–IV with clades V–VIII to assess the effect of imprinting on gene duplication and molecular evolution. Meanwhile, *B* *. rapa* and *T* *. parvula* genes showed a completely different phylogenetic relation between these two clusters. A clade composed of six orthologous sequences from *B* *. rapa* and two sequences from *T* *. parvula* was clustered with clades III and IV (designated as clade B). The phylogenetic position of this clade is close to the base of clades that contain the imprinted genes, implying that *B* *. rapa* and *T* *. parvula* would have experienced independent duplication events during their evolutionary histories. In contrast to sequences in clade B, other *B* *. rapa* and *T* *. parvula* sequences were clustered with each of clade V–VIII. For each numbered clade, a detailed phylogenetic relation was separately investigated using only *A. thaliana* and its close relatives that showed high similarity of sequences (Figure S1 in File S1). *B* *. rapa* and *T* *. parvula* were not included, because these two species were highly diverged from *A. thaliana* causing inaccurate phylogenetic estimations.

### Estimated number of gene duplication in each clade

The result of phylogenetic analyses obtained using a publicly available database indicated that the incidence of gene duplication and genomic imprinting is variable among clades and species. To investigate the variation in gene duplication among more close relatives, orthologous sequences of *A* *. lyrata* ssp. *petraea* and three subspecies of *A* *. halleri* were amplified. The number of sequences obtained for each clade is shown in Table 1. Allelic variants might be included in the count because these species are outbred. Interestingly, there were several duplicated sequences in clades I–IV that contain imprinted genes. In some cases, duplicated sequences clustered to each other and seldom had segregating sites among them (For example, five sequences of *A* *. lyrata* ssp. *lyrata* in clade IV; Figure S2 in File S1) implying recent tandem gene duplication within species. However, the gene duplication does not always occur in orthologous sequences of imprinted genes but is also found in the paralogous sequences of the clade containing known imprinted genes (clade IV in Figure S1 in File S1). In contrast to abundant gene duplications in clades I–IV, there were fewer gene duplications in clades V–VIII. No duplicated gene was found in *A* *. lyrata* ssp. *petraea* . The apparent difference in the gene duplication patterns between clade I-IV and clade V-VIII indicates that different evolutionary mechanisms might contribute to the status of genomic imprinting in these clades.

**Table 1:** Estimated number of duplication in each clade.

	*A. thaliana*	*A* *. lyrata*		*A* *. halleri*			*B* *. rapa*	*T* *. parvula*
		ssp. *lyrata*	ssp. *petraea*	ssp. *gemmifera*	ssp. *tatrica*	ssp. *halleri*		
I	PHE1^♂^, PHE2	6 (4, 2)	5 (4, 1)	2 (1, 1)	1	3 (2, 1)	-	-
II	At5g35120, At1g59920, At1g59930^♀^	3 (1, 1, 1)	3 (1, 1, 1)	3 (1, 1, 1)	3 (1, 1, 1)	3 (1, 1, 1)	-	-
III	AGL34, AGL36^♀^, AGL90	1	2 (2)	3 (3)	3 (3)	3 (3)	-	-
IV	AGL86, AGL92^♂^	5 (4, 1)	4 (3, 1)	1	7 (6, 1)	6 (5, 1)	-	-
V	AGL35	1	1	1	1	1	2 (1, 1)	1
VI	AGL80	1	1	no data	2 (2)	2 (2)	1	4 (1, 1, 1, 1)
VII	AGL46	1	1	1	1	1	2 (1, 1)	2 (1, 1)
VIII	AGL45	1	1	1	1	1	1	1
B	-	-	-	-	-	-	6 (1, 2, 1, 2)	2 (1, 1)

Numbers in parentheses represent a set of sequences with divergence less than 0.1.

♂ paternally expressed gene.

♀ maternally expressed gene.

### Molecular evolution of type I MADS-box genes

**Figure 3:**
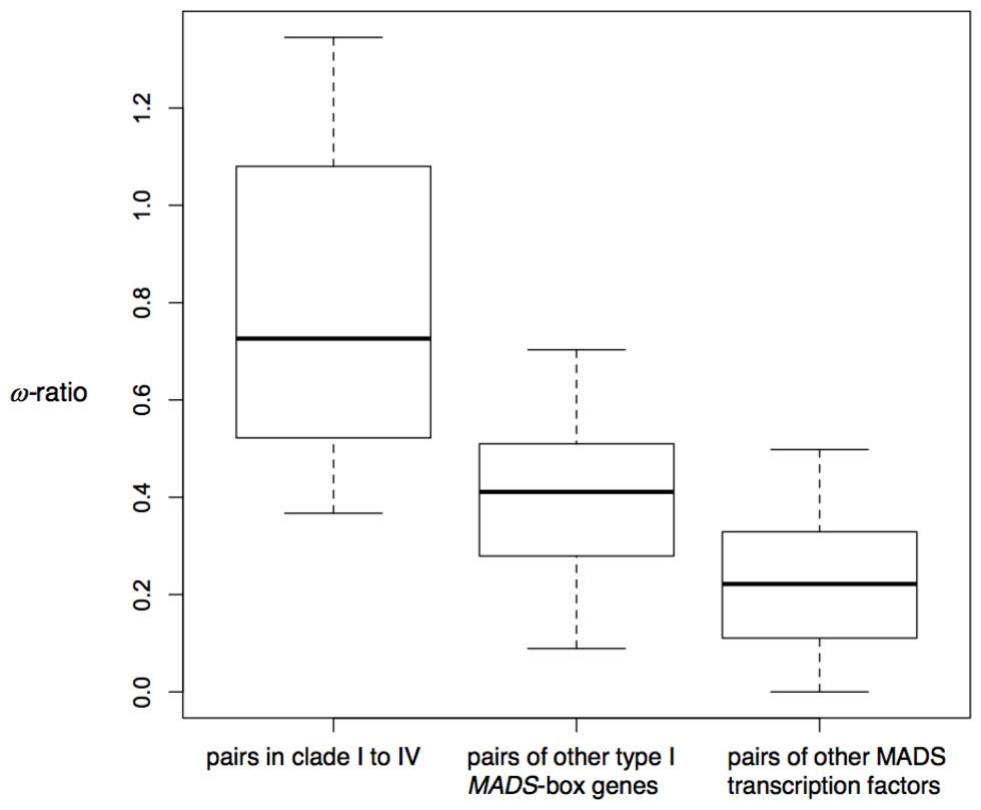
Boxplot representation of pairwise ω-ratio of MADS-box genes between *A. thaliana* and *A. lyrata*. Left; gene pairs in clade I–IV, center; gene pairs in clade V–VIII, and right; gene pairs of other MADS transcription factor family genes. Bold lines represent medians and thin lines of edges of the box represent the first and third quartiles. Lines from whiskers represent minimum and maximum values.

Pairwise ω-ratio between each type I MADS-box gene of *A. thaliana* and *A* *. lyrata* was compared with other MADS transcription factor family genes (Figure 3). Apparent orthologous pairs between the two species were used to estimate the ω-ratio: six pairs of type I MADS-box genes in clades I–IV, 29 pairs of other type I MADS-box genes, and 36 pairs of other MADS transcription factor family genes (type II MADS-box genes and others). A median ω-ratio of orthologous gene pairs in clades I–IV was 0.73, whereas those of other type I MADS-box genes and other MADS transcription factor family genes were 0.41 and 0.23, respectively. A median ω-ratio of gene pairs between *A. thaliana* and *A* *. lyrata* in clades I–IV was significantly higher than those of other type I MADS-box gene pairs and other MADS transcription factor family gene pairs, respectively (*p* = 0.0047 and *p* = 0.0003, respectively; Wilcoxon Rank Sum). This difference suggested that the type I MADS-box gene clades, including imprinted genes, were under positive selection or under relaxed selective constraint.

**Figure 4:**
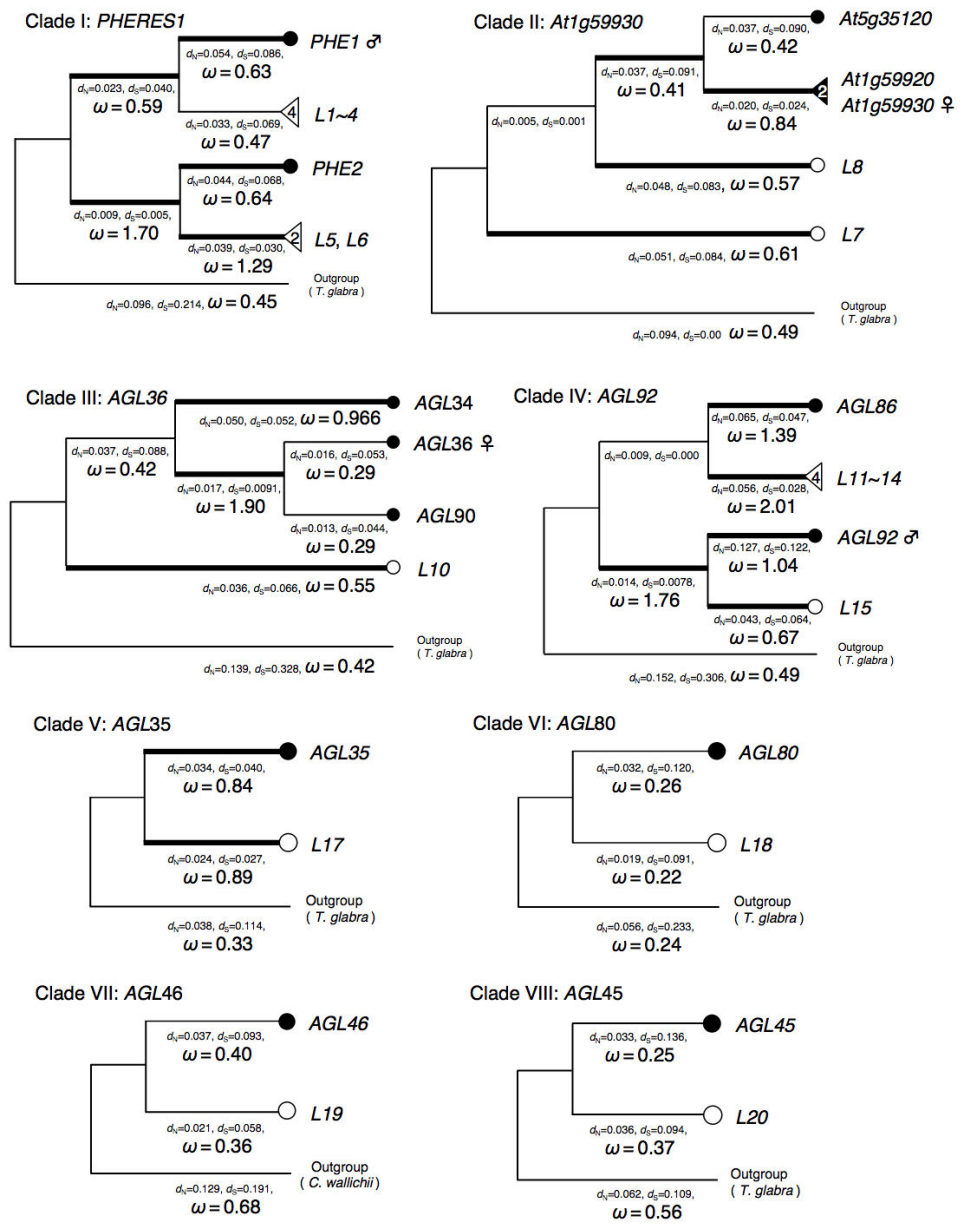
Phylogenetic trees of the type I MADS-BOX genes. For each clade, phylogenetic tree was estimated by Neighbor-joining method with Jukes and Canter’s distance using MEGA5. For convenience, sequences of *A*. *lyrata* were numbered from *L1* to *L20*. Orthologous sequences from *T*. *glabra* and *C*. *wallichii* were used as out-groups for each clade. Imprinting status of *A*. *thaliana* are shown after gene name as follows: ♂; paternally expressed gene, ♀; maternally expressed gene. ω-ratio, *d*_N_, and *d*_S_ values estimated by PAML are shown below the branches. Origin of the species is indicated as follows: Black circles and triangles; *A*. *thaliana*, empty circles and triangles; *A*. *lyrata* ssp. *lyrata*. Triangles represent cluster of recently duplicated genes. Copy number of the cluster is shown in the triangle. Branches with high ω-ratio (ω > 0.5) are shown as thick lines.

To investigate the rate of evolutionary changes on the branches of each clade, computational simulations were conducted using the program package PAML. The estimated ω-ratio and tree topology is shown in Figure 4 with synonymous and non-synonymous distances (*d*_S_ and *d*_N_). Branches in clades containing imprinted genes (I, II, III, and IV) showed relatively high ω-ratio values compared to those in other clades (V, VI, VII, and VIII). Interestingly, some external branches of non-imprinted locus, sister to imprinted genes showed ω > 1 indicative of adaptive evolution (for example clade I and IV). The result suggested that branches in the clade containing imprinted genes evolved faster (higher ω-ratio) than those in the clade without imprinted genes regardless of the imprinting status of the locus. To verify this tendency, a likelihood ratio test was performed using the test statistics 2ΔL = 2(*l*_two-ratio_ -*l*_one-ratio_), where *l*_one-ratio_ and *l*_two-ratio_ are log likelihood values of each model. A one-ratio model assuming a single ω-ratio (*ω*_0_) for all branches was compared with a two-ratio model assuming two ω-ratio: *ω*_1_ for branches in clades containing imprinted genes and branches leading to these clades, and *ω*_2_ for other branches (Table 2). In a two-ratio model, the estimated value of *ω*_1_ was higher than the estimated value of *ω*_0_ (*ω*_1_ = 0.53 and *ω*_2_ = 0.27). The two-ratio model presented a significantly better fit to the data than the one-ratio model with a single ω-ratio for the whole phylogeny (2ΔL = 17.9799; *p* = 0.0000). The significant difference in ω-ratio between the two clades point to the role of different evolutionary forces acting on these clades and the observed differences could be due to the imprinting status of the genes.

**Table 2:** Maximum likelihood parameter estimation for the two models.

	ω*-*ratios		Likelihood ratio test		
	*ω*_0_				
model	*ω*_1_	*ω*_2_	ln*L*	2(ln*L*_two-ratio_ -ln*L*_one-ratio_)	*p*
one-ratio	0.39221		-5211.025458		
two-ratio	0.53408	0.26901	-5202.035533	17.97985	0.0000

## Discussion

### Relationship between gene duplications and imprinted genes

In this study, we observed a positive relationship between number of gene duplication events and presence of imprinted gene in the clades of type I MADS-box gene family. The effect of gene duplications on the evolution of imprinted genes has been firstly discussed in studies of placental mammals [33,34] and later in a genome-wide survey of imprinted genes in plants [4]. In mammals, Walter et al. suggested that imprinted genes have many paralogs that are imprinted or are close to imprinted genes [33]. In a genome-wide survey of *A. thaliana*, significantly higher number of imprinted genes was found in clustered genes than expected by chance [4]. Interestingly, homologous genes of most gene clusters are not always imprinted but include non-imprinted genes. These imprinted genes have a significantly increased numbers of close homologs in comparison to the genome-wide average. From these results, Wolff et al. suggested local gene duplication as a driving force for formation of genomic imprinting [4].

Our finding in this study might, in part, support this hypothesis. The tendency for increased gene duplication in clades containing imprinted genes was detected not only in *A. thaliana*, but also in its close relatives. The question is why and how gene duplication could promote the formation of imprinted genes. There are two ways to understand the relationship between gene duplication and genomic imprinting: (1) gene duplication followed by genomic imprinting, or (2) genomic imprinting leads to gene duplication. In both cases, the first event (gene duplication or genomic imprinting) can change the gene dosage but the direction is opposite. In the former case, gene duplication increases the amount of transcripts and genomic imprinting may control the expression by complete/partial mono-allelic expression. In contrast, in the latter case the newly generated genomic imprinting reduces the expression level. If the reduction is deleterious, a gene duplication that compensates the gene dosage may be selectively advantageous and the frequency of the duplicated mutants may increase. It should be noted that gene duplications could be considered as driving force for the formation of imprinted genes only in the former case.

Previous studies showed that the formation of imprinted genes is tightly associated with the presence of TEs [1,3,4]. The gene duplications and the presence of TEs might not be entirely distinct, but rather, these factors could be linked with each other. Wolff et al. showed a significant enrichment of TEs in the vicinity of imprinted genes [4]. TEs can cause TE-mediated gene duplication that affect evolution of imprinted genes. The possible interplay between gene duplication and presence of TEs during imprinted gene evolution should be analyzed in the future study.

### Molecular evolution of imprinted genes and its homologs

The pattern of molecular evolution of imprinted genes had been analyzed in both plants and placental mammals [4,15–17,35–37]. However, the entire picture of the evolutionary pattern of imprinted genes is still controversial. In plant, some studies suggested natural selection acting on the imprinted gene *MEDEA* (*MEA*) [15,35,36]. Spillane et al. indicated a positive Darwinian selection on an external branch, leading to *A* *. lyrata* *MEA* [15], while Kawabe et al. found high diversity in the *MEA* promoter region and suggested balancing selection acting on the promoter [35]. In contrast, Haun et al. concluded that the Enhancer-of-zeste, orthologous gene of Arabidopsis* MEA*, is under pressure of purifying selection [17]. Recently, molecular evolution of other imprinted genes has been analyzed. The genome-wide survey of imprinted gene suggests rapid evolution of candidate genes relative to other backgrounds [4].

In the previous studies, it has been assumed that the formation of imprinting is the main cause of observed pattern of molecular evolution such as high ω-ratio. Our result might bring a new insight into the pattern of molecular evolution of imprinted genes. The results in this study imply a rapid evolution of not only imprinted genes but also its paralogous genes. For example, ω-ratio of the branch leading to *L5* and *L6* in clade I is higher than 1, while the ω-ratio of branches of *AtPHE1* and *L1 ~ 4* is approximately 0.5. In the case of clade IV, ω-ratio of the branch leading to *L11~14* is higher than 2 (Figure 4) while its sister locus *AGL86* was not imprinted. In addition, model selection test between one-ratio and two-ratio models by PAML suggested the higher ω-ratio of clades I–IV than that of clades V–VIII. These high ω-ratios might not be directly due to genomic imprinting but due to gene duplication followed by neo-functionalization or relaxation of selective constraint. In theoretical studies of gene duplication, the trajectory of duplicated genes and effects of duplications on its molecular evolution have been investigated [38–45]. Gene duplication is a main source of new genes [46] and causes higher evolutionary rate in one or both duplicates [45]. Innan et al. classified gene duplications and its evolutionary trajectory into four categories (categories I to IV) [47]. Most models predict elevations of ω-ratio before fixation of newly derived mutations in duplicates by relaxed selective pressure, positive selection on duplications or pre-duplicational variations, and pseudogenization of duplicates [47]. Although the result in this study did not specify the most suitable model, the homologous genes of imprinted genes in clades I–IV might evolve faster mainly by the effect of gene duplication.

### Possibility of rapid turnover of imprinted status

Another possibility of discordance between imprinted status and rapid molecular evolution is a rapid turnover of imprinted status among orthologous type I MADS-box genes if the imprinted status is a cause of rapid molecular evolution. A comparison of identified imprinted genes of rice, maize and *A. thaliana* suggests that the conservation of imprinted status among these plant species is limited [12]. For example, Luo et al. suggested 165 candidate imprinted loci of rice but only 27 loci have significant sequence homology with the candidate imprinted loci of *A. thaliana* [5]. These limited conservations across species may represent the result of a rapid formation and degradation of imprinted status. The high values of ω-ratio in internal branches observed in this study might imply vestiges of past rapid evolution caused by genomic imprinting and succeeding rapid molecular evolution. It is important to note that the detail of turnover of imprinting status is still unknown because species mentioned above are phylogenetically divergent and the limited conservation of imprinted loci might reflect independent origin of imprinting for each locus. The survey of expression patterns of candidate imprinted genes in closely related species will provide the estimation of turnover rate of imprinting status to test this hypothesis.

## Conclusion

Our results support the view that there exists a relationship between gene duplication and the generation of genomic imprinting. In addition, the clades including imprinted genes tend to evolve faster. However, genomic imprinting is not always the cause of acceleration of molecular evolution. Instead, our results support the view that gene duplication before or after the generation of new genomic imprinting could cause relaxation of selective constraint or non/sub-functionalization, thus leading to increased ω-ratios that were observed in this study. Gene duplication could be one of the driving forces causing evolution of imprinted genes. In the future, analysis of other imprinted genes using *A. thaliana* and closely related species is necessary to test the generality of this hypothesis.

## Supporting Information

File S1 **Table S1, Figures S1-S2.** Table S1. Primer pairs used in this study. Figure S1. Neighbor-joining trees of clades I–VIII. Phylogenetic relationship was estimated with the Jukes and Cantor distance. Bootstrap values (%) were estimated by 500 replications for each clade and shown at corresponding nodes. All trees are shown in a same scale. A distance bar is shown at the bottom. Sequence of each gene excluding alignment gaps or indels was used for estimations. Black circle; *A. thaliana*, red circle; *A* *. lyrata* ssp. *lyrata* , red triangle; *A* *. lyrata* ssp. *petraea* , green circle; *A* *. halleri* ssp. *gemmifera* , open green circle; *A* *. halleri* ssp. *halleri* , green triangle circle; *A* *. halleri* ssp. *tatrica* , empty diamond; *C* *. wallichii* , black diamond; *T* *. glabra* . Figure S2. Location of the homologs in the genome of *A* *. lyrata* ssp. *lyrata* . Each line represents large scaffolds covering the majority of each of the 8 chromosomes of *A* *. lyrata* . The numbered boxes from L1 to L20 representing homologous sequences are identical with sequences in Figure 4. Tandem duplicated sequences are shown in scaffold 1 and 2.
(PPTX)
