# Supplementary material for: Importance of Gene Duplication in the Evolution of Genomic Imprinting Revealed by Molecular Evolutionary Analysis of the Type I MADS-Box Gene Family in Arabidopsis Species
Source: PLoS One. 2013 Sep 5;8(9):e73588. doi: 10.1371/journal.pone.0073588 (PMC3764040; doi:10.1371/journal.pone.0073588)
Supplement: File S1 — Table S1, Figures S1-S2. Table S1. Primer pairs used in this study. Figure S1. Neighbor-joining trees of clades I–VIII. Phylogenetic relationship was estimated with the Jukes and Cantor distance. Bootstrap values (%) were estimated by 500 replications for each clade and shown at corresponding nodes. All trees are shown in a same scale. A distance bar is shown at the bottom. Sequence of each gene excluding alignment gaps or indels was used for estimations. Black circle; A. thaliana, red circle; A . lyrata ssp. lyrata , red triangle; A . lyrata ssp. petraea , green circle; A . halleri ssp. gemmifera , open green circle; A . halleri ssp. halleri , green triangle circle; A . halleri ssp. tatrica , empty diamond; C . wallichii , black diamond; T . glabra . Figure S2. Location of the homologs in the genome of A . lyrata ssp. lyrata . Each line represents large scaffolds covering the majority of each of the 8 chromosomes of A . lyrata . The numbered boxes from L1 to L20 representing homologous sequences are identical with sequences in Figure 4. Tandem duplicated sequences are shown in scaffold 1 and 2. (PPTX) [file pone.0073588.s001.pptx]

## Slide 1
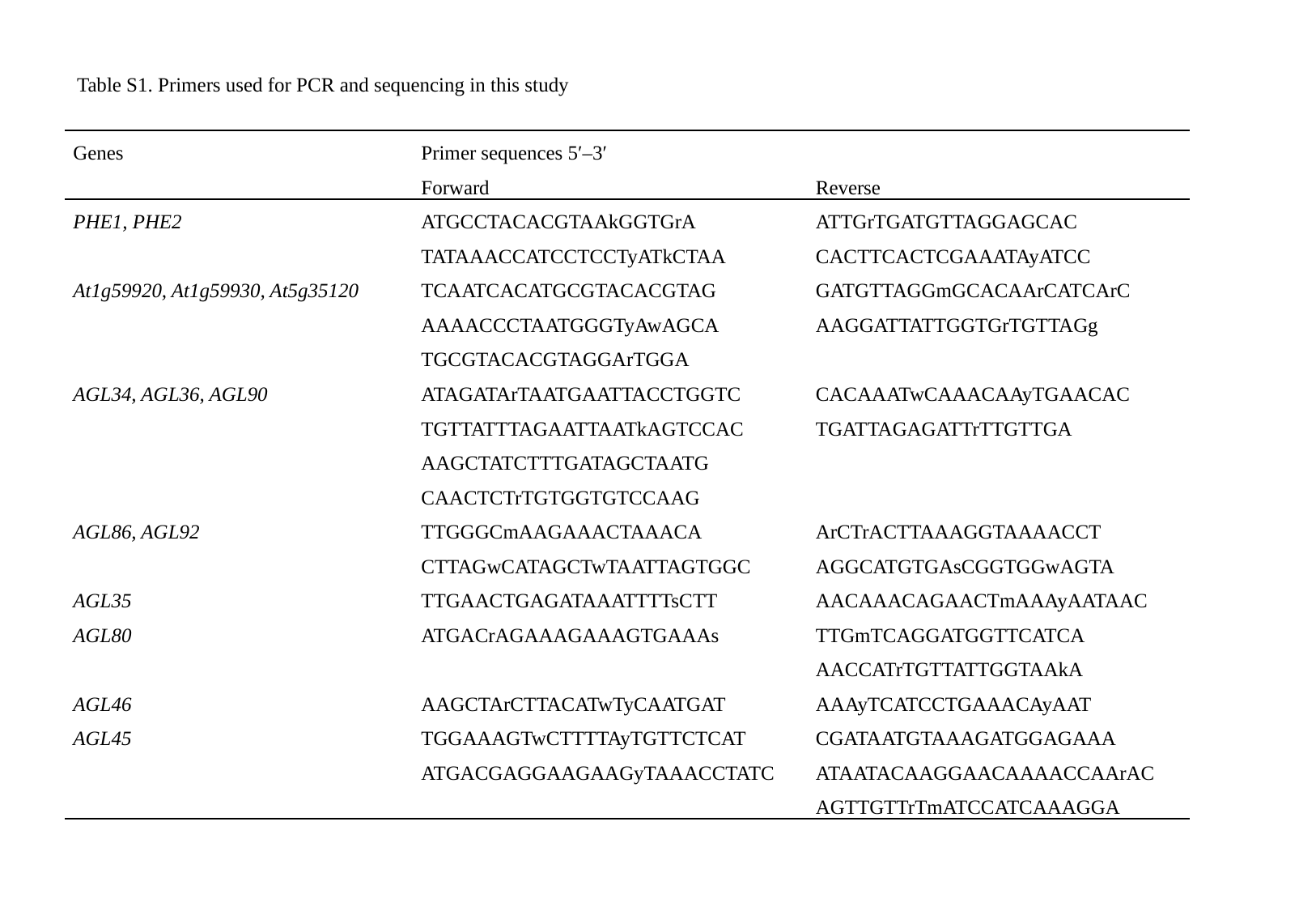

Table S1. Primers used for PCR and sequencing in this study
| Genes | Primer sequences 5′–3′ | |
| --- | --- | --- |
| | Forward | Reverse |
| PHE1, PHE2 | ATGCCTACACGTAAkGGTGrA | ATTGrTGATGTTAGGAGCAC |
| | TATAAACCATCCTCCTyATkCTAA | CACTTCACTCGAAATAyATCC |
| At1g59920, At1g59930, At5g35120 | TCAATCACATGCGTACACGTAG | GATGTTAGGmGCACAArCATCArC |
| | AAAACCCTAATGGGTyAwAGCA | AAGGATTATTGGTGrTGTTAGg |
| | TGCGTACACGTAGGArTGGA | |
| AGL34, AGL36, AGL90 | ATAGATArTAATGAATTACCTGGTC | CACAAATwCAAACAAyTGAACAC |
| | TGTTATTTAGAATTAATkAGTCCAC | TGATTAGAGATTrTTGTTGA |
| | AAGCTATCTTTGATAGCTAATG | |
| | CAACTCTrTGTGGTGTCCAAG | |
| AGL86, AGL92 | TTGGGCmAAGAAACTAAACA | ArCTrACTTAAAGGTAAAACCT |
| | CTTAGwCATAGCTwTAATTAGTGGC | AGGCATGTGAsCGGTGGwAGTA |
| AGL35 | TTGAACTGAGATAAATTTTsCTT | AACAAACAGAACTmAAAyAATAAC |
| AGL80 | ATGACrAGAAAGAAAGTGAAAs | TTGmTCAGGATGGTTCATCA |
| | | AACCATrTGTTATTGGTAAkA |
| AGL46 | AAGCTArCTTACATwTyCAATGAT | AAAyTCATCCTGAAACAyAAT |
| AGL45 | TGGAAAGTwCTTTTAyTGTTCTCAT | CGATAATGTAAAGATGGAGAAA |
| | ATGACGAGGAAGAAGyTAAACCTATC | ATAATACAAGGAACAAAACCAArAC |
| | | AGTTGTTrTmATCCATCAAAGGA |

## Slide 2
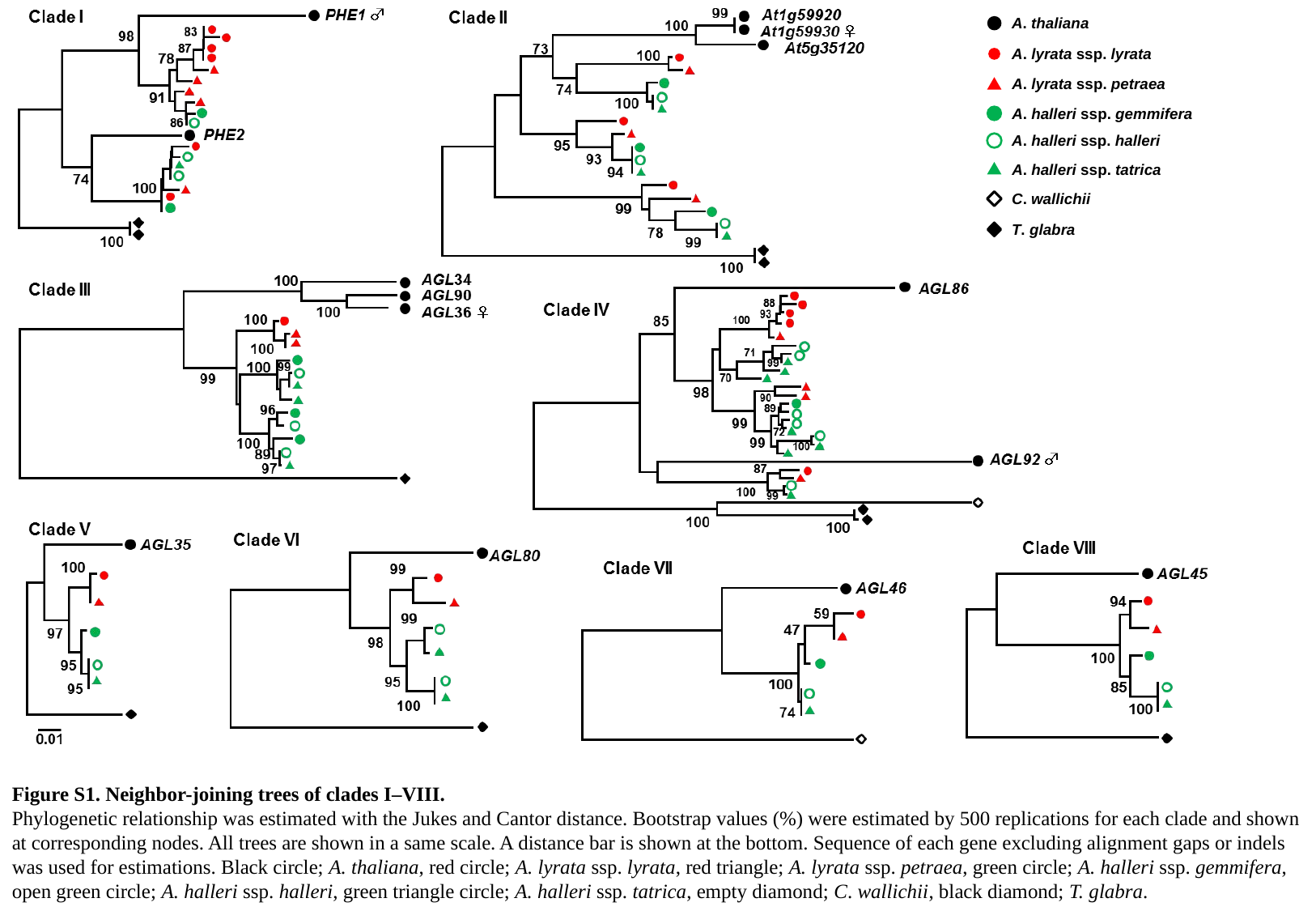

A. thaliana
A. lyrata ssp. lyrata
A. lyrata ssp. petraea
A. halleri ssp. gemmifera
A. halleri ssp. halleri
A. halleri ssp. tatrica
C. wallichii
T. glabra
Figure S1. Neighbor-joining trees of clades I–VIII.
Phylogenetic relationship was estimated with the Jukes and Cantor distance. Bootstrap values (%) were estimated by 500 replications for each clade and shown at corresponding nodes. All trees are shown in a same scale. A distance bar is shown at the bottom. Sequence of each gene excluding alignment gaps or indels was used for estimations. Black circle; A. thaliana, red circle; A. lyrata ssp. lyrata, red triangle; A. lyrata ssp. petraea, green circle; A. halleri ssp. gemmifera, open green circle; A. halleri ssp. halleri, green triangle circle; A. halleri ssp. tatrica, empty diamond; C. wallichii, black diamond; T. glabra.

## Slide 3
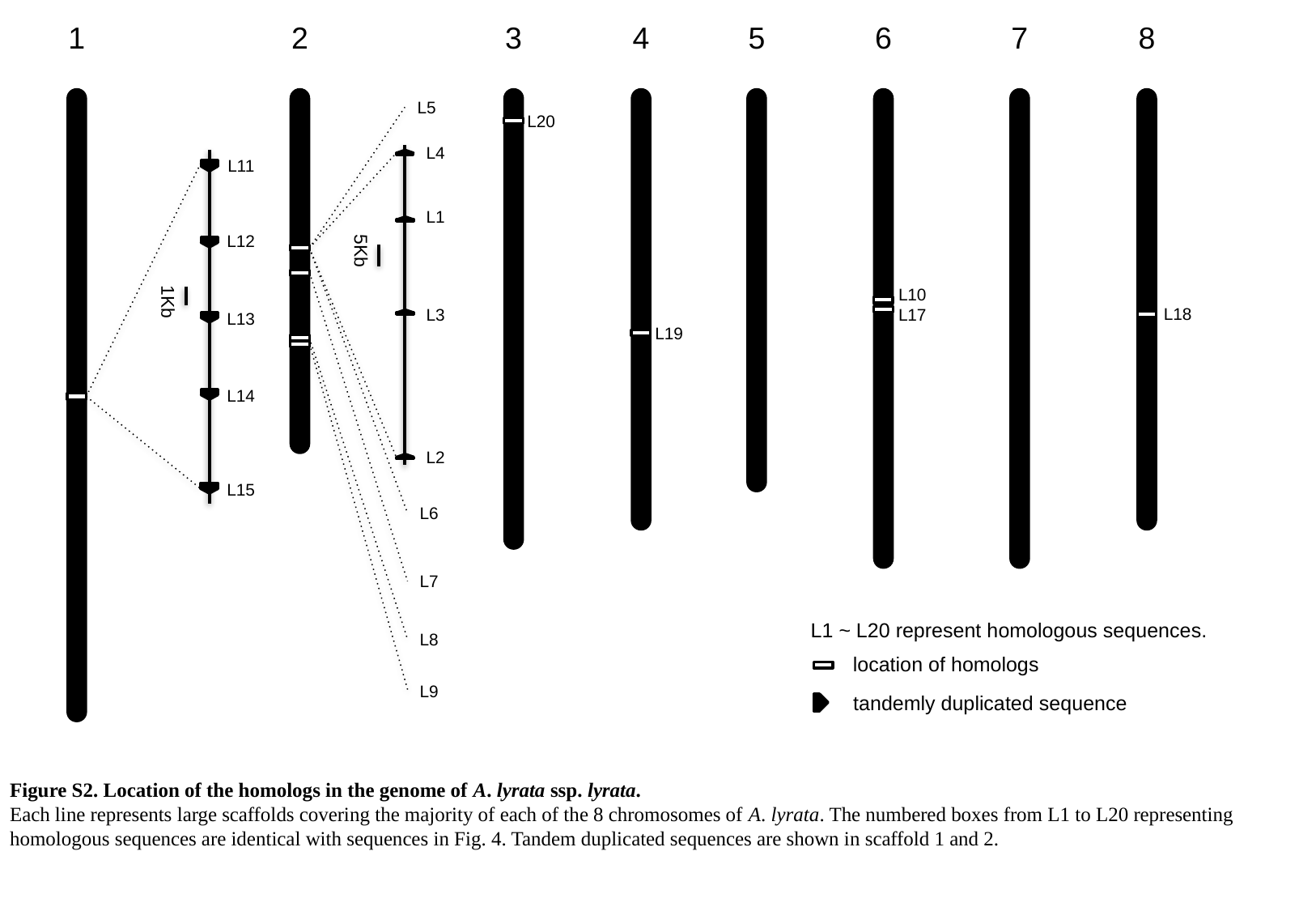

1
L11
L12
1Kb
L13
L14
L15
2
L5
L4
L1
5Kb
L3
L2
L6
L7
L8
L9
3
L20
4
L19
5
6
L10
L17
7
8
L18
L1 ~ L20 represent homologous sequences.
location of homologs
tandemly duplicated sequence
Figure S2. Location of the homologs in the genome of A. lyrata ssp. lyrata.
Each line represents large scaffolds covering the majority of each of the 8 chromosomes of A. lyrata. The numbered boxes from L1 to L20 representing homologous sequences are identical with sequences in Fig. 4. Tandem duplicated sequences are shown in scaffold 1 and 2.
